# Haptoglobin as a novel predictor of visceral involvement and relapse in adult IgAV patients

**DOI:** 10.1007/s10067-025-07363-6

**Published:** 2025-02-14

**Authors:** Matija Bajželj, Nina Visočnik, Katjuša Mrak Poljšak, Matjaž Hladnik, Katja Lakota, Alojzija Hočevar

**Affiliations:** 1 Department of Rheumatology, University Medical Centre Ljubljana, Ljubljana, Slovenia; 2 Faculty of Mathematics, Natural Sciences and Information Technologies, University of Primorska, Koper, Slovenia; 3 Faculty of Medicine, Internal Medicine, University of Ljubljana, Ljubljana, Slovenia; 4 Vasculitis Research Unit, Department of Autoimmune Diseases, Hospital Clínic Barcelona, IDIBAPS, Barcelona, Spain

**Keywords:** Haptoglobin, IgA vasculitis, Transcriptomic data

## Abstract

**Introduction:**

IgA vasculitis (IgAV) can present as skin-limited or systemic disease, which can be severe in adults. Predictive markers for visceral involvement are suboptimal. Considering haptoglobin’s role as an acute phase reactant, we evaluated whether its differential expression in IgAV patients’ skin and leukocytes is also reflected systemically in a larger cohort of adult IgAV patients. Additionally, soluble form of haptoglobin scavenger receptor CD163 was measured in IgAV patient serum.

**Methods:**

We re-analyzed RNA sequencing data from leukocytes and skin biopsies of treatment-naïve adult IgAV patients: (1) IgAV nephritis (*n* = 3), (2) skin-limited IgAV (*n* = 3), and healthy controls (*n* = 3). Haptoglobin serum level was measured in 178, and haptoglobin genotyping was performed in 91 treatment-naïve adult IgAV patients. Serum sCD163 was measured in 60 IgAV patients and 22 HC.

**Results:**

Transcriptomic data of leukocytes and skin of IgAV nephritis patients identified haptoglobin as a hub gene, based on protein–protein interaction network. Haptoglobin serum level was elevated in IgAV patients with nephritis or gastrointestinal involvement compared to other IgAV patients. Patients who relapsed during follow-up had decreased haptoglobin serum level at disease presentation compared to non-relapsing patients. Haptoglobin genotyping did not show differences between genotype groups regarding clinical presentation and laboratory parameters. Serum sCD163 was significantly higher in IgAV nephritis patients compared to HC.

**Conclusion:**

We identified haptoglobin as a novel marker of visceral involvement and relapse in adult IgAV, while sCD163 is linked to renal involvement. Further studies will confirm the clinical utility of haptoglobin as biomarker in IgAV.

**Table:** 

**Key Points** • *Haptoglobin expression is upregulated in leukocytes and skin of adult IgAV with renal involvement.* • *Haptoglobin serum level is elevated in IgAV patients with visceral involvement.* • *Patients with IgAV relapse have lower haptoglobin at disease presentation.*

**Supplementary Information:**

The online version contains supplementary material available at 10.1007/s10067-025-07363-6.

## Introduction

IgA vasculitis (IgAV) is a small-vessel vasculitis characterized by immunoglobulin (Ig) A1-dominant immune complex deposits and typically affects the skin, joints, gastrointestinal tract, and kidneys [1]. IgAV can develop in all age groups. However, in children, it is usually self-limiting, while in adults, IgAV visceral involvement can be associated with poor outcome [2]. Specifically, gastrointestinal tract (GIT) and kidney involvement have an important impact on patient’s short- and long-term prognosis [2–5]. In addition, IgAV relapses affect 15 to 20% of adult patients [2]. Because of this, there is a need for non-invasive biomarkers for early identification and continuous monitoring of potential visceral involvement and development of relapse.

As an immune-mediated inflammatory disease, IgAV is associated with increased serum levels of inflammatory markers, such as C-reactive protein (CRP) and serum amyloid A (SAA) [6, 7]. Recently, we have described another acute phase protein, lipopolysaccharide binding protein (LBP), as a potential predictive biomarker for renal involvement in IgAV [8]. Haptoglobin is a moderate acute-phase protein whose synthesis in hepatocytes, adipocytes, and mesothelial cells is stimulated 2–10 fold by pro-inflammatory cytokines, including interleukin (IL)−6 [9]. Notably, elevated levels of IL-6, TNF (tumor necrosis factor)-α and IL-8 have been reported in patients with IgAV [6]. Haptoglobin is also stored in granulocytes [10] and exhibits immune-modulatory properties, participates in NO inhibition, stimulates tissue repair, and is involved in angiogenesis [11, 12]. Haptoglobin binds with great affinity to free hemoglobin to form soluble hemoglobin-haptoglobin complexes. This allows free hemoglobin to be recycled rather than filtrated through the kidneys, which limits iron loss and at the same time reduces hemoglobin-induced oxidative damage to surrounding tissue [13, 14]. The removal of these complexes is facilitated by the scavenger receptor CD163 mediated endocytosis on monocytes and tissue macrophages in the liver and spleen [12]. Haptoglobin exists in two allelic forms in human population, denoted as Haptoglobin 1 and Haptoglobin 2, which results in three phenotypes: homozygous Haptoglobin 1–1 and Haptoglobin 2–2, and heterozygous Haptoglobin 2–1. Precursor of Haptoglobin 2 was identified as zonulin, a protein that regulates gut permeability and is associated with an increased risk for cardiovascular, autoimmune, diabetic, and infectious disease [11]. So far, serum haptoglobin has been studied in giant cell arteritis (GCA) [15], Takayasu arteritis [16], Behçet disease [17], ANCA-associated vasculitis [18], and Kawasaki disease [19], but association with organ involvement was observed only in GCA [15].

The aim of our prospective study was to investigate the potential role of haptoglobin as a biomarker in IgAV. We evaluated whether the differential expression of haptoglobin in IgAV patients’ skin and leukocytes is also reflected systemically in a larger cohort of adult IgAV patients. In addition, we studied the potential association between CD163 mRNA expression, serum soluble CD163 (sCD163) levels, and haptoglobin phenotype, as well as patients’ clinical characteristics. As the present study demonstrates the potential of haptoglobin as a novel biomarker, future multi-center studies are needed to confirm its clinical utility, while mechanistic studies are required to elucidate haptoglobin’s role in the pathogenesis of IgA vasculitis.

## Methods

### Study design, study subjects, and clinical data collection

We performed a prospective, cross-sectional study and presented our findings in original research article. The study included one hundred and seventy-eight patients diagnosed with IgAV and followed at the University Medical Center Ljubljana (UMCL) between February 2015 and July 2022. All patients included in our study were immunosuppressive treatment naïve. The diagnosis of IgAV was clinical, according to the 2012 revised International Chapel Hill Consensus Conference Nomenclature of Vasculitides definition [1]. All included IgAV patients also fulfilled the 2010 European League Against Rheumatism/Paediatric Rheumatology International Trials Organisation/Paediatric Rheumatology European Society (EULAR/PRINTO/PRES) classification criteria and had histologically-proven IgAV [20]. Patients without histologically-proven IgAV were excluded from the study. Prior to sample and clinical data collection, subjects signed an informed consent. The study was approved by the Slovene National Medical Ethics Committee (#159/07/13, #99/04/15, #65/01/17, and #0120–121/2021/3).

Clinical data collected at diagnosis were age, sex, body mass index (BMI), smoking status, duration of clinical symptoms to diagnosis, past medical history, including past (occurred within 1 month before IgAV diagnosis and already cured) and concurrent infections, and IgAV-related signs and symptoms. Disease activity was determined using Birmingham vasculitis activity score version 3 (BVAS-3) [21]. The definitions of purpura above waistline, GI involvement (and its severity), and renal involvement (and its severity graduation) used in this study have been reported in detail previously [22]. Healthy controls (HC) were investigated at the Department of Rheumatology, UMCL for the presence of systemic autoimmune rheumatic diseases (SARDs) and immunosuppressive treatment as an exclusion criteria.

### Laboratory parameters

Erythrocyte sedimentation rate (ESR), C-reactive protein (CRP), complete blood count with differential, basic biochemistry panels including electrolytes, creatinine, urea, serum protein electrophoresis, serum IgA, IgM, IgG levels, complement components C3 and C4, and urine analysis were determined in routine diagnostics. The serum concentration of haptoglobin and serum amyloid A (SAA) was determined by immunonephelometry (BN Prospec System, Siemens).

### Re-analysis of RNA sequencing data

Differentially expressed genes (DEGs) in the deposited data of skin (PRJNA1017657) and leukocytes (PRJNA1136414) of IgAV-with renal involvement (IgAVN) (*n* = 3), patients with skin-limited IgAV (sl-IgAV) (*n* = 3), and age-and sex-matched HC (*n* = 3) were identified as already performed in [8]. DEGs were defined with shrunken log2 fold change ≥ │1│, *p*-adj < 0.05. Protein–protein interaction analysis was performed in STRING database.

### ELISA measurement

After blood withdrawal, blood clotting was allowed for 30 min, samples were centrifuged at 3000 × g for 5 min, and sera were aliquoted and stored at − 20 °C until further analysis. Soluble CD163 was measured in sera from 59 treatment-naïve adult IgAV patients and 22 age-/sex-matched HC using human CD163 Quantikine ELISA Kit (R&D Systems) according to manufacturer instructions.

### Haptoglobin genotyping

In blood samples from 91 patients with IgAV, haptoglobin gene was analyzed for the presence of alleles Haptoglobin 1 and Haptoglobin 2 using the TaqMan genotyping assay [23]. Samples for genotyping were collected from IgAV patients from 2017 to 2022. The sequence of primers and probes used was 5′-CGTTATTAGGAGGAGCTGTTGCT-3′ (forward primer), 5′- CACACCAGTAAGAGCAGAAGAG-3′ (reverse primer), VIC-ATTCTCAGAACAAGAGGCA-3′ (probe A binding equally to intron 4 of haptoglobin 1 and intron 6 of the haptoglobin 2 allele), and FAM-CTCAGAACCAGAGGCA-3′ (probe C binding to a site in intron 4 of haptoglobin 2) [23].

### Statistical analysis

The data were analyzed with GraphPad Prism version 8.0. and IBM SPSS Statistics 29. To assess whether the data followed a normal distribution, both the Shapiro–Wilk and Kolmogorov–Smirnov tests were employed. Data that were normally distributed were expressed as means. For comparing two groups, a two-tailed parametric t-test was used, and for comparisons involving more than two groups, a one-way analysis of variance (ANOVA) was applied. For data that were not normally distributed, the median and interquartile range (IQR) were reported. In these cases, the Mann–Whitney U test was used for two-group comparisons, while the Kruskal–Wallis test was applied for comparisons involving multiple groups. To account for multiple comparisons, Tukey’s test (when variances were unequal) or Dunn’s post hoc test was performed. Cross tabulations, Chi-square tests, and Fisher’s exact tests were utilized to compare categorical variables across different phenotype groups. *p*-values of < 0.05 were regarded as statistically significant.

## Results

### Re-analysis of the leukocyte and skin transcriptomic profiles from all IgAV patients, IgAVN, sl-IgAV, and HC

**Fig. 1:**
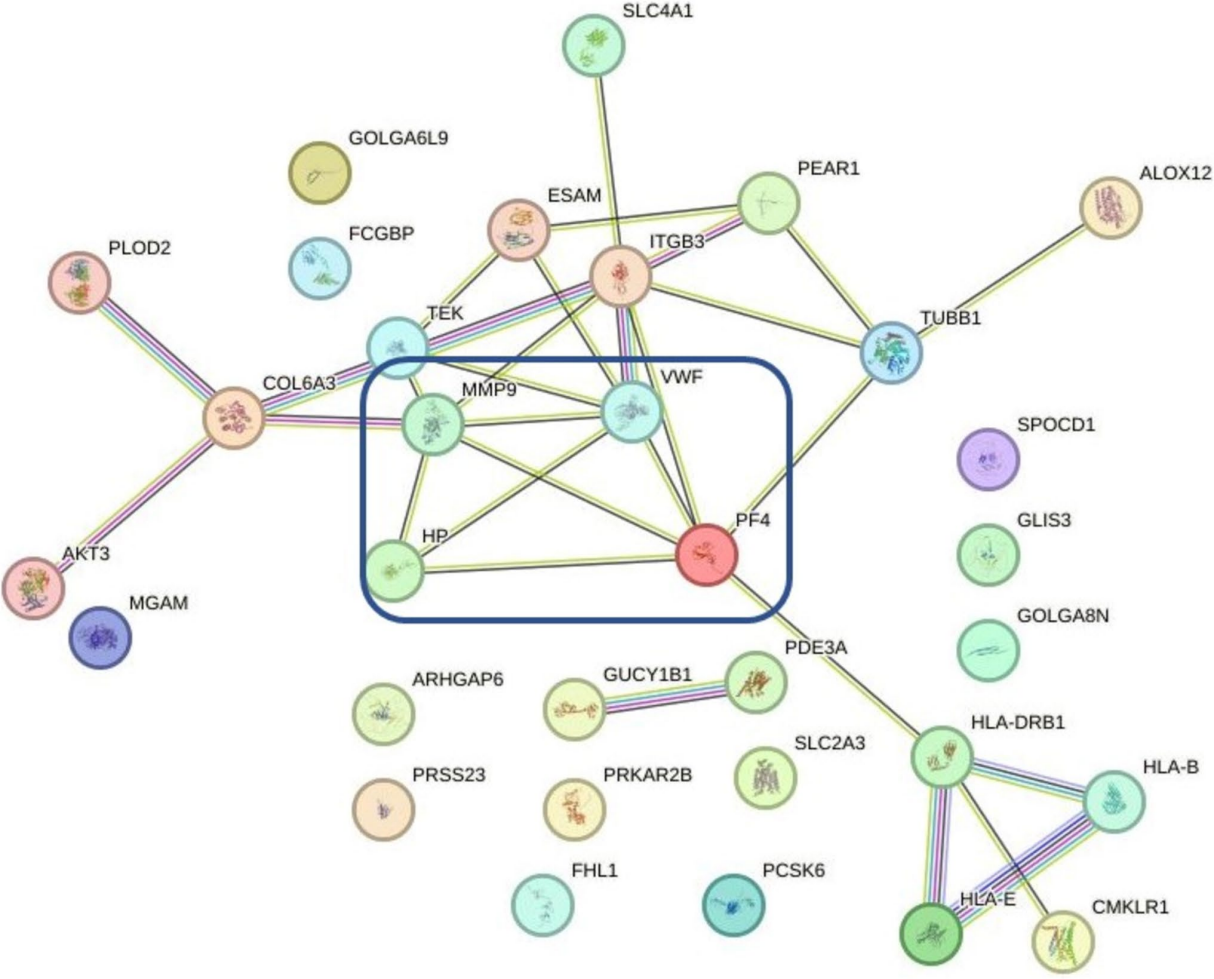
STRING network presents common DEGs in skin and leukocytes of IgAVN patients. HP, MMP9, VWF, and PF4 were identified as hub genes. Nodes represent genes and edges represent interactions between them. DEGs, differentially expressed genes; IgAV, immunoglobulin A vasculitis; IgAVN, IgAV-renal involvement; HP, haptoglobin; MMP9, matrix metalloproteinase-9; VWF; Von Willebrand factor; PF4, platelet factor 4

Re-analysis of bulk sequencing data from six adult treatment-naïve IgAV patients (demographic and clinical data in Table S1) as compared to HC revealed 174 DEGs in the leukocytes and 49 in the skin, with majority of genes being upregulated. When analyzing only IgAVN patients as compared to HC, we identified 234 DEGs in leukocytes and 507 DEGs in the skin, while in sl-IgAV patients, we identified 121 DEGs in leukocytes and 46 DEGs in the skin (Table 1). Thirty-two overlapping genes between skin and leukocytes were identified in IgAVN patients and among them 4 hub genes haptoglobin, matrix metalloproteinase-9 (MMP9), Von Willebrand factor (VWF), and platelet factor 4 (PF4/CXCL4) (Fig. 1). In IgAVN patients, haptoglobin was identified among the top twenty-five most overexpressed genes in skin (logFC = 5.52; *p*-adj = 3.58 × 10^−6^) and leukocytes (logFC = 2.9; *p*-adj = 3.91 × 10^−7^) (Figure S1), while in sl-IgAV was not among DEGs for the skin (logFC = 6.59 × 10^−7^; *p*-adj = 0.172) or for the leukocytes (logFC = 0.628; *p*-adj = 0.0725). Differential expression of haptoglobin only in patients with IgAVN but not in patients with sl-IgAV suggested its potential association with renal involvement.

**Table 1:** Number of DEGs (|log2(FC)|≥ 1 and *p*-adjusted value (*p*-adj) ≤ 0.05) in IgAV patients vs. healthy controls (HC), IgAVN vs. HC, and sl-IgAV vs. HC

Comparison (A vs. B)	Number DEGs in leukocytes	Number DEGs in the skin
IgAV vs. HC	174	49
IgAVN vs. HC	234	507
sl-IgAV vs. HC	121	46

*IgAV* immunoglobulin A vasculitis, *IgAVN* IgAV-renal involvement, *IgAV_S* IgAV-skin-limited disease, *HC* healthy controls, *DEGs* differentially expressed genes

### Haptoglobin serum level and its associations with clinical manifestations

**Fig. 2:**
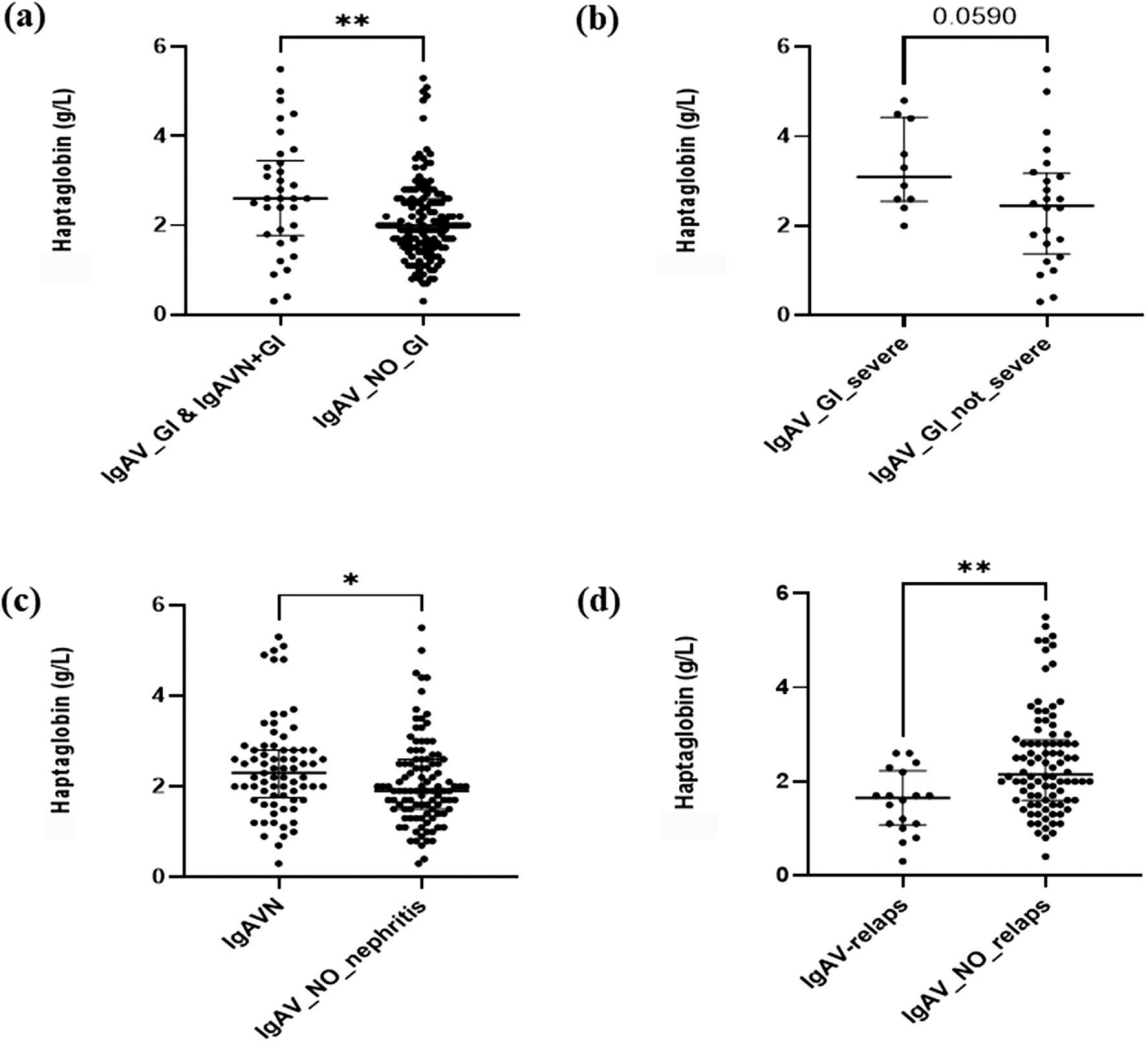
Associations of haptoglobin serum levels with clinical presentation. **a** Haptoglobin serum level was significantly higher in patients with GI involvement as compared to those without. **b** Patients with severe GI involvement had increased serum haptoglobin levels as compared to other IgAV_GI patients. **c** Haptoglobin was significantly higher in IgAVN patients as compared to those without renal involvement. **d** Patients with relapse had significantly lower haptoglobin values at disease presentation as compared to non-relapsed IgAV patients. *p*-values were calculated using Mann–Whitney U test. Data are expressed as medians (Q25–Q75) of each group. *p ≤ 0.05, **p ≤ 0.01, ***p ≤ 0.001. IgAV, immunoglobulin A vasculitis

Haptoglobin serum level was measured in 178 adult IgAV patients (110 males, 68 females, median (IQR) age of 62.2 (46.8–72.2) years). All patients presented with skin purpura (purpura above the waistline 52.8%, necrotic in 51.1%). Renal, gastrointestinal, and joint involvement were present in 41.0%, 19.1%, and 26.4%, respectively. Concurrent renal and gastrointestinal tract involvement were present in 17 patients (9.6%). Ten out of 34 patients (29.4%) had severe gastrointestinal involvement, while 19 out of 73 (26.0%) had severe renal involvement (Table 2). The pre-treatment haptoglobin serum concentration exceeded the upper limit of reference (normal range 0.5–2.0 g/l) in 88 (49.4%) cases. The median (IQR) concentration in IgAV patients was 2.0 (1.6–2.7) g/l. IgAVN patients had significantly higher serum haptoglobin concentrations compared to those without renal involvement (median (IQR) 2.3 (1.75–2.8) vs. 1.9 (1.5–2.6) g/l, *p* = 0.018) (Fig. 2a). IgAV_GI patients also had significantly higher haptoglobin level as compared to those without GI involvement (median (IQR) 2.6 (1.78–3.45) vs. 2.0 (1.5–2.6) g/l, *p* = 6.3 × 10^−3^) (Fig. 2b). Patients with severe GI involvement had higher haptoglobin level compared to other IgAV_GI patients (median (IQR) 3.1 (2.55–4.43) vs. 2.45 (1.38–3.18) g/l, *p* = 0.059) (Fig. 2c).

**Table 2:** Demographic, laboratory, and clinical characteristics of IgAV patients included in serum haptoglobin measurement

Characteristics	IgAV (*N* = 178)
Age	62.2 (46.8–72.2)
Sex	110 M, 68 F
BMI*	27.9 (23.8–32.9)
Symptom duration (day)*	7.5 (5–14)
Symptoms and signs *N* (%)	
General symptoms	28 (15.7)
Fever	16 (9.0)
Weight loss	15 (8.4)
Skin purpura	178 (100)
Purpura above waistline	94 (52.8)
Skin necroses	91 (51.1)
Joint involvement	47 (26.4)
GI involvement	34 (19.1)
Renal involvement	73 (41)
Concurrent infection	29 (16.3)
Prior infection	59 (33.1)
Diabetes type II	39 (21.9)
Arterial hypertension	90 (50.6)
Heart failure	34 (19.1)
Chronic kidney failure	29 (16.3)
BVAS* (Q25–Q75)	6 (2–12.3)
ESR* (mm/h)	33 (16.8–53.3)
CRP* (g/l)	21 (6–46)
White cells* (10^9^/l)	7.85 (6.5–9.6)
Number of lymphocytes (10^9^/l)	1.56 (1.11–2.00)
Number of neutrophils (10^9^/l)	5.54 (4.40–7.19)
IgA* (g/l)	4.10 (2.57–5.40)
IgG* (g/l)	13.3 (11.2–15.1)
IgM* (g/l)	0.815 (0.59–1.16)
C3* (g/l)	1.32 (1.15–1.47)
C4* (g/l)	0.28 (0.23–0.33)

General symptoms; fever, weight loss, loss of appetite; *IgAV* immunoglobulin A vasculitis, *HC* healthy controls, *M* male, *F* female, *BVAS* Birmingham vasculitis activity score, *ESR* erythrocyte sedimentation rate, *CRP* C-reactive protein (CRP), *Ig* immunoglobulin, * median (IQR), *BMI* body mass index

### Low serum haptoglobin at diagnosis is associated with relapsing IgAV

IgAV relapse was defined as described in detail in [2]. Follow-up data were available for 110 out of 178 patients (61.8%). During a median 17.1 (IQR 12–26) month follow-up, relapses were recorded in 18 patients. 14/18 (76.9%) patients had a single organ relapse, and 4/18 had a multi-organ relapse. Relapsing IgAV patients had at baseline significantly lower haptoglobin levels compared to non-relapsing patients (median (IQR) 1.65 (1.08–2.23) vs. 2.15 (1.6–2.88) g/l, *p* = 1.5 × 10^−3^) (Fig. 2d).

### Correlations between haptoglobin and routine laboratory parameters

**Fig. 3:**
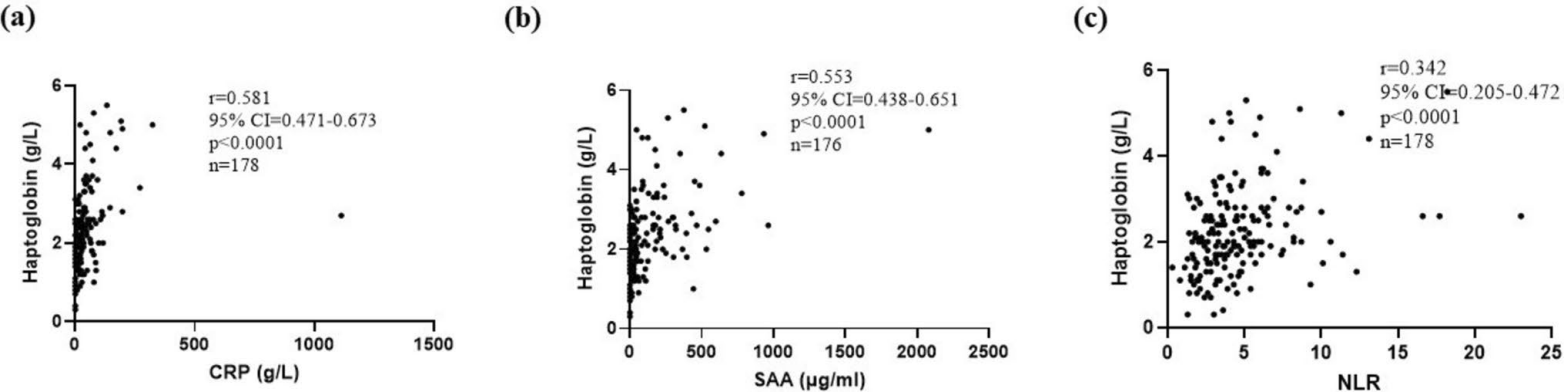
Correlation between haptoglobin and CRP concentration (**a**), SAA concentration (**b**), and NLR ratio (**c**) in IgAV patients. Shown are Spearman correlation coefficient (*r*), 95% confidence interval, *p*-value, and numbers of included patients. CI, confidence interval; IgAV, immunoglobulin A vasculitis; NLR, neutrophil to lymphocyte ratio; SAA; serum amyloid A

Significant positive correlations were observed between haptoglobin and other inflammatory markers such as ESR (rs = 0.536, *p* < 1 × 10^−3^), CRP (rs = 0.581, *p* < 1 × 10^−3^) (Fig. 3a), and SAA (rs = 0.553, *p* < 1 × 10^−3^) (Fig. 3b). Haptoglobin serum level positively correlated with neutrophil (rs = 0.342, *p* < 1 × 10^−3^) and white blood cell (WBC) count (rs = 0.271, *p* < 1 × 10^−3^) and neutrophil-to-lymphocyte ratio (NLR) (rs = 0.345, *p* < 1 × 10^−3^) (Fig. 3c), as well as complement components C3 (*r*_*s*_ = 0.302, *p* < 1 × 10^−3^) and C4 (rs = 0.308, *p* < 1 × 10^−3^). Negative correlation was observed between haptoglobin serum level and serum albumin (rs = − 0.434, *p* < 1 × 10^−3^).

### Haptoglobin genotypes in adult patients with IgAV

Scatterplots of TaqMan fluorescence signals yielded distinct clusters corresponding to Haptoglobin 1–1, Haptoglobin 2–1, and Haptoglobin 2–2 genotypes (Figure S2). Genotype frequencies in IgAV cohort were 16.5%, 48.4%, and 35.2% for Haptoglobin 1–1, Haptoglobin 2–1, and Haptoglobin 2–2, respectively (Table S2). Genotype distribution did not deviate from the Hardy–Weinberg equilibrium. Statistical analysis did not reveal significant differences in clinical characteristics and laboratory values between the different genotype groups of patients (Table S2).

### CD163 skin mRNA expression and serum sCD163 serum level

**Fig. 4:**
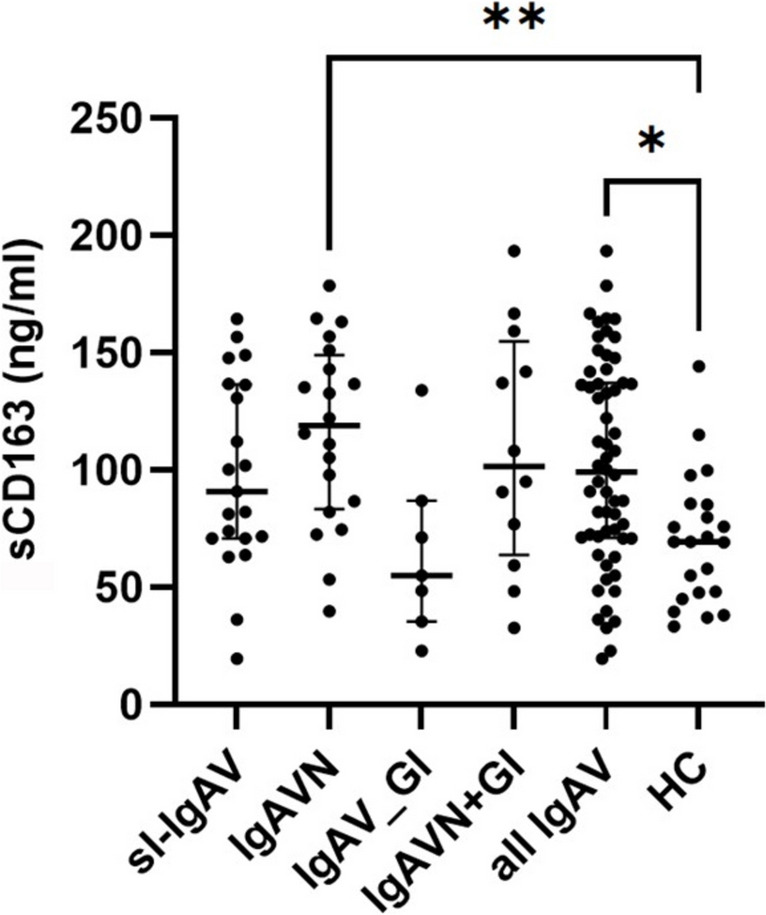
sCD163 was significantly higher in IgAVN patients as compared to IgAV_GI patients. *p*-values were calculated using Mann–Whitney U test. Data are expressed as medians (Q25–Q75) of each group. *p ≤ 0.05, **p ≤ 0.01, ***p ≤ 0.001. IgAV, immunoglobulin A vasculitis; sl-IgAV, skin-limited IgAV; IgAVN, IgAV with renal involvement; IgAV_GI, IgA with gastrointestinal involvement (GI); IgAVN + GI, IgAV with GI and renal involvement; HC, healthy controls

CD163 was significantly upregulated (FC = 2.19, *p*-adj = 2.18 × 10^−2^) only in the skin of IgAVN patients, as revealed by our RNAseq data. To investigate also systemic dysregulation, we measured (sCD163) in serum of 60 patients and 22 HC (Table S3). Serum sCD163 was significantly higher in all IgAV patients compared to HC (median (IQR) of 99.3 (71.1–137.1) vs. 69.5 (47.1–85.4) ng/ml, *p* = 1.5 × 10^–3^) and in IgAVN patients compared to HC (median (IQR) of 119.0 (83.4–149.1) vs. 69.5 (47.1 –85.4) ng/ml, p = 4.7 × 10^−3^) (Fig. 4). No significant association between sCD163 and serum haptoglobin was observed (rs=-0.0233, p=0.869) (Figure S3).

## Discussion

Results of our study support the potential role of haptoglobin as a stratification biomarker in IgAV. In a large cohort of treatment naïve adult IgAV patients, we found a significant elevation of serum haptoglobin levels in individuals with renal and/or GIT involvement compared to those without visceral involvement. Elevated serum haptoglobin levels in IgAVN patients confirmed our transcriptomic data showing increased haptoglobin mRNA expression in leukocytes and skin of IgAVN as compared to HC. Moreover, haptoglobin levels were increased in patients with severe GI involvement compared to non-severe GI involvement, further suggesting the role of haptoglobin as a marker of IgAV severity.

Increased serum levels of haptoglobin in IgAV patients with gastrointestinal involvement are in line with studies reporting an increase of other acute phase reactants such as SAA, CRP, and procalcitonin, suggesting more intense inflammatory response in patients with GI involvement [24–26]. We observed elevated SAA (*p* = 0.017 and *p* = 0.052) and CRP (*p* = 0.001 and *p* = 0.003) in patients with GI and also renal involvement compared to those without respective organ involvement. These findings align with an increase of pro-inflammatory cytokines, such as IL1β, IL-4, IL-6, and IL8, found in IgAV with or without kidney or GI involvement [27]. Elevated levels of haptoglobin in IgAV patients with renal involvement could also be due to subsequent renal insufficiency, as it is linked to a pro-inflammatory response and is more pronounced in advanced stages of chronic kidney disease [28]. Moreover, elevated haptoglobin levels in plasma and urine have been also identified in several different murine models of acute-kidney injury [29].

Our data indicate that lower levels of serum haptoglobin at disease presentation are associated with relapse, which is in line with Hočevar et al. reporting that patients with IgAV limited to skin relapse more frequently than patients with additional organ involvement [2]. During follow-up, patients were monitored for persistent abnormal urinalysis, a marker of response to treatment, as persistent abnormal urinalysis was associated with baseline immunomodulatory treatment, as reported in Hočevar et al. [2]. However, we did not measure haptoglobin serum levels during follow-up and were therefore not able to compare haptoglobin serum levels at disease presentation with clinical symptoms during follow-up.

As haptoglobin genotype has been linked to immune-mediated diseases, we genotyped a portion of our patients and found that the frequency of different genotype groups corresponded to the haptoglobin genotype distribution in Europe (Hp1-1 8–16%, Hp2-1 41–51%, Hp2-2 34–51%) [30] and general Slovenian population (Hp1-1 8%, Hp2-1 52%, Hp2-2 39%) [31]. In addition, no significant difference was found in clinical characteristics between patients of different genotype groups.

Interestingly, our RNA-seq analysis of skin biopsy samples from IgAVN patients revealed a significant upregulation of the monocyte and macrophage scavenger receptor CD163, which is a key receptor in the endocytosis of the hemoglobin-haptoglobin complex. CD163 is a marker of alternatively activated, anti-inflammatory and tissue repair type of macrophages [32], which might explain the upregulation of CD163 mRNA in the affected skin of our IgAVN patients. Though macrophage infiltrates are present in IgAV patients’ skin [33], it has yet to be determined which macrophage subtype (M1 or M2) is more abundant in skin of IgAV patients. Although all IgAV patients presented with increased serum sCD163 levels compared to HC, substantially higher sCD163 was found in IgAVN patients compared to those without renal involvement (*p* = 0.073), which is consistent with our RNA seq data, showing an increase only in patients with renal involvement. While IL-6 is the major stimulus for haptoglobin synthesis [34], CD163 expression is chiefly dependent on IL-10 [35]. Kuret et al. showed elevated levels of IL-6 in IgAV patients, while IL-10 level was not changed compared to HC [6]. Furthermore, metalloproteinase 17 (ADAMTS-17, named also TNF-α converting enzyme—TACE) cleaves the membrane bound CD163 upon inflammatory stimuli or oxidative stress [36, 37], and measuring ADAMTS-17 might reveal the mechanism behind increased sCD163 in serum. Urinary sCD163 has been identified as a biomarker of disease activity in patients with ANCA-associated vasculitis with glomerulonephritis [38–40], suggesting that measuring it might also be of interest in IgAVN.

However, our study has some limitations. As the median age of IgAV patients included in our cohort was 62.2 years, comorbidities might contribute to elevated haptoglobin levels in some patients. Follow-up haptoglobin serum levels and haptoglobin levels at relapse could provide additional information of disease activity and response to treatment. The haptoglobin genotype was determined in a limited number of IgAV patients to ensure consistency in the DNA isolation method used for blood samples.

To our knowledge, this is the first study analyzing haptoglobin and serum sCD163 and their association with organ involvement in adult IgAV patients. Our study suggests the routine measuring of haptoglobin serum levels in clinical practice as a novel biomarker for developing IgAV visceral involvement and relapses. The advantage of serum haptoglobin as a marker lies in its routine use in clinical practice, making it readily available to assist in the management of patients with IgAV. Additionally, based on our study, serum sCD163 could be used for stratifying patients with GI and renal involvement. All our findings warrant confirmatory studies.

## Supplementary Information

Below is the link to the electronic supplementary material.

Supplementary file1 (DOCX 215 KB)

## Data Availability

All data have been uploaded to a publicly available database (SRA#PRJNA1017657 and PRJNA1136414) and are also available by written request to the corresponding author.
