# Supplementary material for: Haptoglobin as a novel predictor of visceral involvement and relapse in adult IgAV patients
Source: Clin Rheumatol. 2025 Feb 14;44(4):1665–73. doi: 10.1007/s10067-025-07363-6 (PMC11993498; doi:10.1007/s10067-025-07363-6)
Supplement: Supplementary file 1 — Supplementary file1 (DOCX 215 KB) [file 10067_2025_7363_MOESM1_ESM.docx]

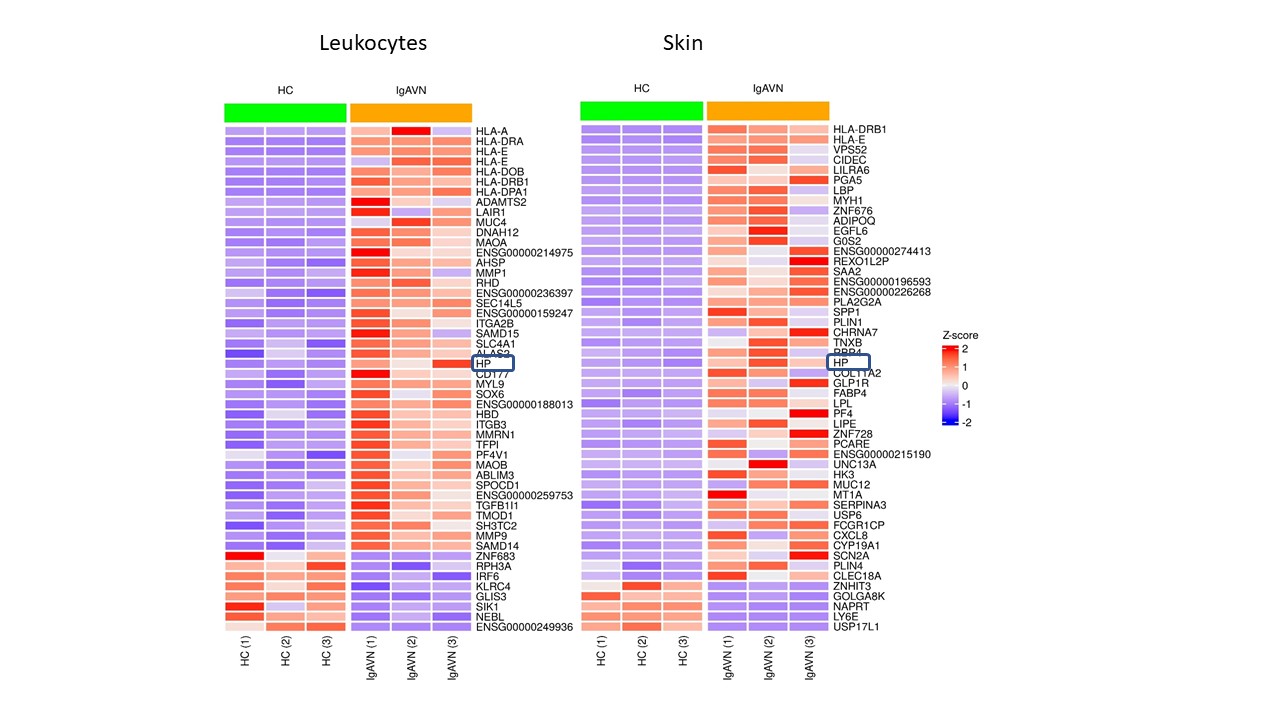


**Figure S1** Heatmap represents the 50 most differentially expressed genes with the largest lfc values across IgAVN patients and HC. The lfc values were adjusted using the apeglm method, and the genes are sorted by these values. Red colours indicate overexpression and blue colours indicate underexpression. Haptoglobin was identified among the most highly differentially overexpressed genes in leukocytes **(a)** and skin **(b)** of IgAVN patients as compared to HC. HP, haptoglobin; IgAV, immunoglobulin A vasculitis; IgAVN, IgAV-renal involvement; HC, healthy controls.


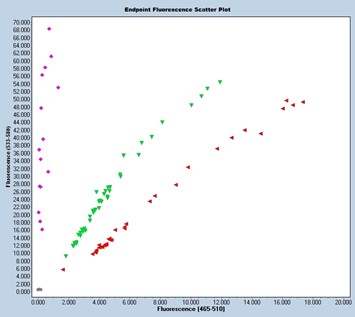


**Figure S2:** Scatterplot of TaqMan fluorescence signal.


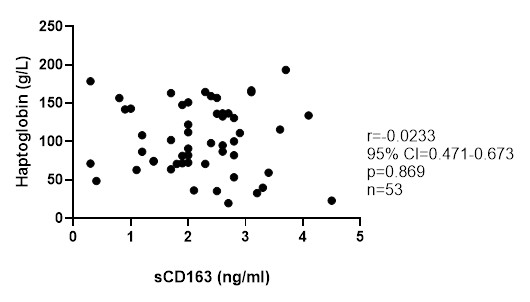


**Figure S3** Correlation between haptoglobin and sCD163 in IgAV patients. Shown are Spearman correlation coefficient (r), 95% confidence interval, p value, and numbers of included patients. CI, confidence interval; IgAV, immunoglobulin A vasculitis.

**Table S1** Demographic and clinical characteristics of IgAV patients included in RNA sequencing

|  | Patients | HC |  |
| --- | --- | --- | --- |
| Age  Median (Q25–Q75) | 66.5 (56.6-76.7) | 53 (48–57.5) |  |
| sex | 5F; 1M | 3F |  |
| Skin symptom duration | 5.5 (3.5-38) days |  |  |
| Clinical information | 6 skin purpura; 2 necrotic, 1 bullous skin lesions  3 patients had renal involvement  1 concurrent, 2 patients had recent infection |  |  |

IgAV, Immunoglobulin A vasculitis; HC, healthy controls; M, male; F, female;

Table S2: Characteristics of adult IgAV patients according to their Hp genotype:

| **Characteristics** | **Haptoglobin genotype** | | | |
| --- | --- | --- | --- | --- |
|  | **Hp1-1** | **Hp2-1** | **Hp2-2** | ***p*-value** |
| Number | 15 (16,5%) | 44 (48,4%) | 31 (35,2%) |  |
| Age | 49,4 (34,85- 77,15) | 59,65 (40,95-69,55) | 61,4014 (49,6-71,6) | 0,470 (Kurskal-Wallis) |
| Male gender | 8 (53,3%) | 30 (68,2%) | 19 (59,4%) | 0,528 (Chi-Square) |
| **Clinical manifestations** | | | | |
| Skin involvement | 8 (53,3%) | 21 (47,7%) | 19 (59,4%) | 0,603 (Chi-Square) |
| Joint involvement | 1 (6,7%) | 6 (13,6%) | 3 (9,4%) | 0,821 (Fischer) |
| GIT involvement | 4 (26,7%) | 9 (20,5%) | 7 (21,9%) | 0,890 (Fischer) |
| Severe GIT involvement | 2 (13,3%) | 2 (4,5%) | 3 (9,4%) | 0,391 (Fischer) |
| Kidney involvement | 8 (53,3%) | 18 (40,9%) | 12 (37,5%) | 0,583 (Chi-Square) |
| Severe renal involvement | 3 (20%) | 3 (6,8%) | 5 (15,6%) | 0,238 (Fischer) |
| AKI or worsening of CKD (KDIGO definition) | 2 (13,3%) | 2 (4,5%) | 2 (6,3%) | 0,449 (Fischer) |
| eGF (MDRD) | 91 (57,5-91) | 81 (61-91) | 86 (59-91) | 0,541 (KW) |
| Creatinine (mmol/L) | 76 (62-100,5) | 82,5 (72-100,5) | 73 (62,25-94,5) | 0,257 (KW) |
| New onset arterial hypertension | 2 (13,3%) | 3 (6,85%) | 3 (9,4%) | 0,619 (Fischer) |
| Hematuria | 10 (66,7%) | 25 (56,8%) | 15 (46,9%) | 0,420 (Chi-Square) |
| Glomerular Hematuria | 0 (0%) | 5 (11,4%) | 1 (3,1%) | 0,309 (Fischer exact test) |
| Macrohematuria | 2 (13,3%) | 3 (6,8%) | 0 (0%) | 0,111 (Fischer) |
| Proteinuria | 6 (40%) | 24 (54,5%) | 17 (53,1%) | 0,609 (Chi-Square) |
| Nephrotic-range proteinuria | 3 (20%) | 3 (6,8%) | 2 (6,3%) | 0,251 (Fischer) |
| eDP (mg/day) | 106 (71-559,5) | 159,5 (91,75- 744) | 164,5 (78-1011,5) | 0,985 (KW) |
| Selective proteinuria | 2 (13,3%) | 13 (29,5%) | 9 (28,1%) | 0,549 (Fischer) |
| Non-selective proteinuria | 1 (6,7%) | 4 (9,1%) | 2 (6,3%) | 1,000 (Fischer) |
| Tubular proteinuria | 3 (20%) | 3 (6,8%) | 5 (15,6%) | 0,201 (Fischer) |
| BVAS | 14 (2,5-17) | 6 (2,25-12) | 6 (4-12,75) | 0,347 (KW) |
| Relaps | 1 (6,7%) | 6 (13,6%) | 2 (6,3%) | 0,624 (Fischer) |
| **Laboratory values** | | | | |
| ESR | 27 (14-38,5) | 41,5 (24,75 - 61) | 34,5 (21,25-66) | 0,402 (KW) |
| CRP | 38 (5-94) | 25,5 (6,5-48,25) | 23,5 (9-46,5) | 0,262 (KW) |
| SAA | 239 (6,2-430) | 39 (10,45-122,25) | 44,5 (17,975-165,75) | 0,405 (KW) |
| Haptoglobin | 2,5 (1,75-3,05) | 2,2 (1,9 -3,1) | 2 (1,4-2,575) | 0,172 (KW) |
| Albumin | 34 (30,5-37,5) | 36 (33,5-42,5) | 35,5 (30,25-39) | 0,073 (KW) |
| Leukocytes | 8,8 (6,95 - 9,5) | 7,95 (6,35-10,25) | 7,7 (6,425-9,675) | 0,820 (KW) |
| Neutrophils | 6 (4,21-7) | 5,51 (4,47 -7,445) | 5,32 (4,2-7,295) | 0,675 (KW) |
| Lymphocytes | 1,02 (0,815 -2,15) | 1,5 (1,1425-1,9225) | 1,535(0,8775-1,8825) | 0,200 (KW) |
| NLR | 5 (2,5-7,6) | 4,2 (3 -5,3) | 3,65 (2,5-6,1) | 0,558 (KW) |
| Hb (g/L) | 137 (115,5-140,5) | 134 (121-145) | 128,5(112,5-143,75) | 0,153 (KW) |
| Thrombocytes | 243 (205,5-380,5) | 278,5(207,5-317,75) | 257 (212-328,5) | 0,977 (KW) |
| Number of patients with elevated IgA in serum | 5 (33,3%) | 23 (52,3%) | 18 (56,3%) | 0,325 (Chi-Square) |
| IgA concentration | 2,47 (2,31- 3,41) | 4,01 (2,4075-6,115) | 3,985(2,235-4,9475) | 0,673 (KW) |
| IgG concentration | 11,3 (9,725-14,75) | 13,85(11,425-7,025) | 13,3(11,025-15,825) | 0,232 (KW) |
| IgM concentration | 1,07 (0,63-1,555) | 0,795(0,6175-1,2975) | 0,81 (0,3925-1,005) | 0,534 (KW) |
| C3 | 1,39 (1,155-1,63) | 1,355(1,1475-1,545) | 1,33 (1,1325-1,5375) | 0,784 (ANOVA) |
| C4 | 0,29 (0,23-0,37) | 0,27 (0,23-0,3275) | 0,27 (0,2-0,31) | 0,703 (ANOVA) |

**Table S3: Demographic, laboratory and clinical characteristics of IgAV patients included in serum sCD163 measurement**

| **Characteristics** | **IgAV (N=60)** |
| --- | --- |
| Age | 64.8 (49.5-71.1) |
| Sex | 41 M, 19 F |
| BMI* | 29.2 (24.7-32.4) |
| Symptom duration (day)* | 7 (4.25-14) |
| Symptoms and signs N (%) |  |
| General symptoms | 11 (18.3) |
| Fever | 4 (6.7) |
| Weight loss | 8 (13.3) |
| Skin purpura | 60 (100) |
| Purpura above waistline | 38 (63.3) |
| Skin necroses | 30 (50.0) |
| Joint involvement | 8 (13.3) |
| GI involvement | 19 (31.7) |
| Renal involvement | 31 (51.7) |
| Concurrent infection | 12 (20.0) |
| Prior infection | 19 (31.7) |
| Diabetes type II | 15 (25.0) |
| Arterial hypertension | 32 (53.3) |
| Heart failure | 15 (25.0) |
| Chronic kidney failure | 10 (16.7) |
| BVAS* (Q25–Q75) | 8.5 (2.5-14.0) |
| ESR* (mm/h) | 36 (17.0-54.0) |
| CRP* (g/l) | 21 (6.25-55.3) |
| White cells* (10^9^/l) | 8.8 (7.4-10.3) |
| Number of lymphocytes (10^9^/l) | 1.58 (1.1-1.99) |
| Number of neutrophils (10^9^/l) | 6.05 (4.90-7.90) |
| IgA* (g/l) | 4.23 (2.77-5.33) |
| IgG* (g/l) | 12.3 (9.94-14.9) |
| IgM* (g/l) | 0.75 (0.44-1.07) |
| C3* (g/l) | 1.35 (1.19-1.50) |
| C4* (g/l) | 0.28 (0.23-0.33) |

General symptoms: fever, weight loss, loss of appetite. IgAV, Immunoglobulin A vasculitis; HC, healthy controls; M, male; F, female; BVAS, Birmingham vasculitis activity score; ESR, Erythrocyte sedimentation rate; CRP, C-reactive protein (CRP); Ig, immunoglobulin; * median (IQR); BMI body mass index
